# Ultra-processed products industry operating as an interest group

**DOI:** 10.11606/s1518-8787.2020054002127

**Published:** 2020-10-28

**Authors:** Aline Brandão Mariath, Ana Paula Bortoletto Martins

**Affiliations:** I Câmara dos Deputados Departamento Médico BrasíliaDF Brasil Câmara dos Deputados . Departamento Médico . Brasília , DF , Brasil; II Universidade de São Paulo Faculdade de Saúde Pública Programa de Pós-Graduação em Nutrição em Saúde Pública São PauloSP Brasil Universidade de São Paulo . Faculdade de Saúde Pública . Programa de Pós-Graduação em Nutrição em Saúde Pública . São Paulo , SP , Brasil; III Universidade de São Paulo Núcleo de Pesquisas Epidemiológicas em Nutrição e Saúde São PauloSP Brasil Universidade de São Paulo . Núcleo de Pesquisas Epidemiológicas em Nutrição e Saúde . São Paulo , SP , Brasil

**Keywords:** Food Industry, Conflict of Interest, Public Policy, Food Regulation, Government Regulation

## Abstract

The participation of the ultra-processed products industry in efforts to reduce obesity and diet-related non-communicable diseases has been questioned, especially because there is evidence of its interference in policy-making processes. This article builds on the Collective Action Theory and the literature of political science to discuss the role of this sector as a special interest group that uses its significant economic power to influence government decisions in its favor. In Brazil, its participation occurs mainly with industry associations. However, it has not yet been established whether their interests prevail in the decision-making process. It has been suggested that research should be carried out to determine the degree of success of their actions, identifying the conditions associated with the convergence of policy results with their interests and indicating to what extent civil society organizations are able to make public interests override private ones.

## INTRODUCTION

Nowadays, there is no doubt about the influence of the ultra-processed products industry on food environments and its relationship with the increase in obesity and diet-related non-communicable diseases. This phenomenon is mainly attributed to the accelerated increase in the consumption of these products. Companies in this sector have a great influence over individual attitudes, perceptions, and preferences and over the food supply determining its availability, quality, and price. Furthermore, these companies apply elaborate marketing strategies to promote their products and to shape social norms and beliefs related to food ^1^ . In general, large national and multinational companies usually stand out among other players for driving food decisions in a more direct manner ^2^ .Considering the wide reach of leading food companies, their participation in public health efforts to make food environments healthier has been encouraged. However, this role as part of the solution is controversial, since government actions to improve food systems can affect the cost of production, sales growth, and profits. These measures must be properly contextualized and go beyond self-regulatory initiatives to be sufficiently effective ^2^ . In this sense, it is necessary to recognize that the adoption of regulatory measures by the public sector has been proven essential to protect and to promote the health of population and to adequately address the problem of obesity and diet-related non-communicable diseases ^5^ .

Some reports in scientific literature of public health highlight the participation of the ultra-processed products industry in policy-making, influencing legal and regulatory measures in its favor, or even preventing the establishment of rules that negatively affect its activities ^3 , 7^ .

This article aims to discuss the role of the ultra-processed products industry as a special interest group that uses its significant economic power to intervene in governmental decisions. Although this issue has received more and more attention from the point of view of public health, its discussion based on the perspective of political science is still in the early stages.

This essay is based on Mancur Olson’s Collective Action Theory and the narrative review of national and international literature on interest groups. Evidence regarding the role of the ultra-processed products industry in policy-making was collected from articles published in scientific journals indexed in PubMed and SciELO databases, as well as from the Brazilian federal government and industry associations official websites.

## DEFINITIONS: INTEREST GROUPS, PRESSURE GROUPS, SPECIAL INTEREST GROUPS AND LOBBYING

Several definitions for interest groups exist. Some are more comprehensive and include all types of groups, regardless of their activities, degrees of organization, and cleavages. Others are more restricted and their focus is limited to those who present demands to public authorities. When they become politically active to protect themselves or to promote their interests, interest groups can be called “pressure groups” or “special interest groups”, reflecting the activity that characterizes their participation ^13^ . The term “pressure group” carries more negative connotations than the term “interest group”, and it seems to apply only to sectional groups – those that represent the self-interest of a particular economic or private social group in a society and whose members are restricted to them. Groups can also be considered primary, when their fundamental purpose is to engage in lobbying activities. They are considered secondary when they act politically only if necessary ^16 , 17^ .

Despite the small differences between the numerous definitions, there is a consensus among scholars: interest or pressure groups are those who are in contact with policy-makers to persuade them, influencing public policy in their favor. They can be composed of traditional associations, institutional interest groups, or organizational interest groups ^13^ . Institutional interest groups are not necessarily organizations with associated members, according to the traditional meaning of an interest group. They may comprise public or private institutions, including individual companies. Organizational interest groups may include associations representing their members ^15^ .

With regard to the participation of companies as interest groups, these can be considered a special interest group ^18^ and they usually act directly or by representatives whenever there is a perception that public authority decisions can interfere with their activities ^13^ . According to Olson ^19^ , special interests are highly organized, display disproportionate political power over other organizations, and tend to prevail over unorganized interests of large groups. Thus, small groups composed of large businesses use their capacity for organized and voluntary action to create active lobbying, exert pressure and then obtain political advantages. The main definitions relating to interest groups are summarized in the Box .

**Box:** Summary of definitions and types of interest groups.

Definitions	
Pressure groups	Interest groups that contact decisionmakers of the government to influence policy-making processes in their favor. This term usually applies to sectional groups (which represent the self-interest of an economic or social group and whose members are restricted to them).
Special interest groups	Highly organized interest groups, usually with disproportionate economic (and, consequently, political) power over other organizations.
**Types**	
Primary	Interest groups whose fundamental purpose is to engage in lobbying activities.
Secondary	Interest groups whose fundamental purpose is not political, but which can act in such a manner if necessary.
Institutional	They comprise public or private institutions (including individual companies).
Organizational	They comprise trade associations representing their members.

The concepts and theories described heretofore apply directly to the ultra-processed products industry, especially because it is a concentrated sector on a global scale, with a select group of transnational corporations with great economic power being dominant. This can be regarded as a special interest group, which can be primary, in the case of companies considered individually, and secondary, when they operate represented by their industry associations.

Lobbying is the most usual activity of interest groups in pluralistic systems, in which several organizations that claim the representation of their own interests seek to influence decision-making in public policy ^20^ . This is a multifaceted process that includes information collection, preparation of policy drafts, definition of strategies for defense of such drafts, search for allies, among other activities. It also involves the exercise of pressure, but only in the last stages of lobbying. The lobbyist, despite representing special interests, has specialized information and technical and political knowledge, and is often useful in defining legislation and regulations ^21^ . Although legal lobbying may be positive, as it closes the gap between policy results and the preferences of organized interests, it can have undesirable consequences when there is a great imbalance of power between groups. Those who are stronger and more organized are overly privileged, and may call public interest into question ^20^ .

## INEQUALITY OF REPRESENTATION AND RESOURCES AMONG INTEREST GROUPS

Interest groups are central to political representation, the public policy agenda, and the results of public policies. However, there are important biases in the representation system. Privileged groups, such as business groups, are usually overrepresented in the policy arenas. Public interest groups, which mainly seek the collective good (which when achieved does not benefit its members or activists in a selective or material manner), often face more difficulty to get organized and are usually underrepresented ^22^ . Moreover, the use of influence over political authorities in modern democracies requires a range of capabilities that includes overcoming problems related to collective action, resource mobilization, expertise development, monitoring of policy processes, coordinating actions with other players and acting in various contexts ^23^ .

Promoting interests to decision-makers depends to a large extent on the availability of resources. Undoubtedly, money is the most important one, since it enables the defense of interests and consequently increases the effectiveness of interventions by the groups ^20 , 24^ . Large companies have an advantage on this point: often, their organizational slack allows them to carry out activities that do not have an immediate effect on their profits. In addition, as they have more financial resources, they are able to employ more sophisticated political strategies ^18^ .

There are numerous resources other than money that can also be used, with emphasis on legitimacy or affinity with the preferences of public opinion, the size of the social segment represented, the proportion of the segment actually being profiled behind the lobbies, as well as the characteristics of the lobbyists ^20^ . It is important to note that the business community does not necessarily intervene politically in the same way or with the same degree of frequency or intensity, strategically directing its resources to places and situations that will produce more efficient results ^24^ .

Access to policy arenas is a necessary condition to exert influence on the agenda and the decision-making process, even if by itself it is not sufficient. The supply of resources is important because state institutions and interest groups maintain an interdependent relationship, since they are not able to achieve their political objectives autonomously. However, it has been observed that access to one policy arena provides access to others, in a cascading and cumulative effect. Therefore, more resourceful players can dominate multiple arenas, in what is called “privileged pluralism” ^22^ .

For Mancuso and Gozetto ^20^ , if there were a balance between the active groups, legal lobbying could provide positive contributions to both the interest groups involved and the decision makers, contributing to the legitimization of the political system. According to pluralists, interest groups can contribute to democracy as they supply specialized information to the government, induce the inclusion of important items in the policy agenda, and improve the quality of public debate by bringing their opinions to the media ^17^ . However, resources are not widely available and distributed equitably among interest groups and, therefore, access to policy-making processes and the chances of influencing them are not equal ^18 , 24^ . In fact, no interest group – such as trade unions or public interest groups – is able to compete with the substantial business resources ^18^ . Therefore, the inequality of access and participation in the policy-making processes originates from the inequality of representation and resources. Moreover, although these inequalities reflect the general bias of the distribution of power, they may simply not be replicating it, but reinforcing it ^16^ .

Another issue to be considered, highlighted by Coen et al. ^18^ , is the relationship of power between the State and companies, named by the authors as “structuralism”: States depend fundamentally on companies, because they need the resources and revenues generated by them. Thus, if on the one hand the government can control the companies, on the other, it can also be controlled and dominated by them, in what is called the Capture of the State. The study about the relationships between companies and states raises questions about the effectiveness of government, democracy, and distribution of power in modern society. This is mainly because organized groups that hold more power can dominate and directly help in creating inequities ^18 , 24^ . Therefore, despite being part of the political system, interest groups, by promoting biased benefits in favor of some segments and to the detriment of others, can undermine the fundamental objectives of a society. It should be noted that, although the need to regulate the activities of interest groups is defended, even if it affected their access, it would not necessarily affect their power of influence or reduce inequality ^15^ .

## OBJECTIVES, STRATEGIES, AND CONSEQUENCES OF THE ACTIONS OF INTEREST GROUPS

Interest groups do not pursue the direct exercise of power, but directly or indirectly influence policy-making on specific issues in their interest. Their interaction with public policy-makers usually occurs by lobbying. Lobbying activities do not always seek immediate influence on the decision-making process, and can be employed only to facilitate access to decision makers and build a relationship with government representatives with the intention of obtaining future benefits ^15^ .

The participation of interest groups in public policy processes can occur in several of their stages, from the agenda setting to the formulation and implementation of policies ^17 , 22^ . It is known that these groups often act in coalition or competition in certain areas, employing wide variety of tactics to shape public policies ^24 , 25^ . However, analyzing the causal effects of their interventions is a very complex task, considering the multiplicity of players, resources, and strategies involved, as well as the fact that most actions happen away from public scrutiny.

Despite the difficulty in proving the cause-and-effect relationship between lobbying and policy results, especially due to economic, sociocultural, and historical circumstances that also interfere in the calculation of the decision maker, it is possible to measure the success of the lobbyist ^20^ . Mancuso ^26^ considers that there is political success when the content of the decision converges with the business position. This can occur both in situations where public sector decisions improve the *status quo* and when a decision that would lead to its detriment is not made. Political failure is when the content of the public sector decision differs from the corporate preference. This can occur in two cases: first, when the corporate sector fights against a decision that would worsen the *status quo,* but the decision is made anyway; and second, when the corporate sector defends a decision that would improve the status *quo* , but is not made by the government.

In this context, it can be affirmed that the political action of the corporate sector can have two objectives: 1) improve their *status quo,* which requires an offensive attitude; and 2) maintain or prevent the worsening of their status *quo,* in a defensive attitude ^26^ . Thus, interest groups can act offensively and proactively, to gain advantage in public policy, or in a defensive and reactive manner, seeking to mitigate unfavorable issues. Especially regarding defensive attitudes, Hacker and Pierson ^23^ highlight a strategy widely used by interest groups: encouraging nonaction by government representatives, since this does not attract the attention of voters and the public, and cannot be attributed to a particular policy-maker.

Generally, there are different preferences within interest groups when it involves the employment of resources, the search for access to political arenas, and the definition of targets and pressure points. Corporate interest groups usually prioritize the use of insider resources – such as information and relevant expertise in the decision-making process – and act in less visible arenas, especially when the political objective is to influence decision-making ^17 , 22^ .

With regard to the ultra-processed products industry, its defense of interests is not necessarily limited to approaching decision makers. When engaged in corporate political activity, the sector employs multiple strategies, among which are: the production and dissemination of information favorable to its activities; the distribution of incentives, including financial ones, to politicians, political parties, and decision makers; fostering public opinion favorable to the industry; the destabilization of individuals or groups who criticize or oppose its products or practices; the destabilization of individuals or groups who advocate policies that can negatively affect its business; and the use or threat of use of lawsuits, either to stop government decisions that are unfavorable to it, or to intimidate its opponents ^12^ . Recently, it has also been demonstrated the “policy dystopia” model, developed to describe the corporate political activity of the tobacco industry – widely recognized for its intense action to undermine public health efforts to reduce smoking-related diseases on the population – can also be applied to the ultra-processed products industry ^27 , 28^ .

## EVIDENCE OF THE PARTICIPATION OF ULTRA-PROCESSED PRODUCTS INDUSTRY AS AN INTEREST GROUP IN BRAZIL

In Brazil, there is still an important knowledge gap regarding the attempts of the ultra-processed products industry to influence food and nutrition public policies. From the academic point of view, we are only aware of analyses about the process to regulate food advertising by the Brazilian Health Regulatory Agency (ANVISA), initiated in 2005 and which resulted in the Resolution of the Directors Board (RDC) No. 24/2010. According to Henriques et al. ^29^ , the private sector did not hide its position contrary to the preliminary version of the regulation and its preference for maintaining the *status quo.* Baird ^30^ examined the political action of corporate interest groups in this process. Their evident intervention in ANVISA and in the Executive and Legislative Branches to influence the results of public policy was intensified after the publication of the regulation in question. For the author, the privileged access of lobbyists, facilitated by the great economic power of the industry, contributed to the drafting of a softer version of the regulation. As a result, in its final draft, elements related to advertising aimed at children that had been included in the initial proposal were excluded, making it less restrictive ^29^ .

Notably, there was no direct manifestation – favorable or not – of any ultra-processed products corporation during the formulation of this policy. The participation of the private sector occurred exclusively in an indirect manner, by the *Associação Brasileira das Indústrias da Alimentação* (ABIA – Brazilian Association of Food Industries) and the *Associação Brasileira das Indústrias de Refrigerantes e de Bebidas não Acoólicas* (ABIR – Brazilian Association of Soft Drink and Non-alcoholic Beverage Industries) ^29 , 31^ . This corroborates the literature that indicates that collective action is advantageous, especially when there are common interests at stake or the players involved are threatened by regulatory policies. These policies affect the entire industrial sector and tend to trigger a higher level of conflict, often encouraging affected players both to act individually and to form coalitions to confront those who defend interests different from theirs ^14 , 19 , 21 , 26 , 32^ .

Currently, there is plenty of empirical evidence on the intense participation of the ultra-processed products industry in two ongoing regulatory processes. In the first case, a process initiated in 2014 by ANVISA aimed to establish new rules for the nutritional labeling of industrialized foods and drinks. The sector has been represented by both ABIA and many other associations gathered in the *Rede Rotulagem* (Labeling Network). These players have used several strategies to shape the debate and to favor the adoption of a regulation that has minor effect on consumers’ choices and, therefore, is more favorable to the sector: the traffic light labeling system. We can also mention the use of a legal strategy, where ABIA obtained an injunction from the judiciary to extend the Technical Public Consultation initiated by ANVISA in 2018, which can be interpreted as an attempt to delay the decision-making process ^33^ . The disclosure of the Preliminary Report on the Regulatory Impact Analysis of Food Labeling ^34^ , based on the evaluation of evidence and contributions generated in the Technical Public Consultation, pointed to the preference for the frontal warning labeling model highlighting the presence of excess critical nutrients. It is expected that the ultra-processed products industry will reinforce its opposition and expand the use of strategies to try to stop the regulatory process and/or influence the Agency’s final decision. The second case refers to the consequences of Decree No. 9,394 of May 30, 2018 ^35^ , which withdrew part of the tax benefits granted through tax credits on industrialized products to companies that produce soft drink concentrates in the Manaus Free Trade Zone. This was one of the measures adopted by the Brazilian Government to compensate for the decrease in tax collection generated after the truckers’ strike, which was terminated with the State’s commitment to reduce the final price of diesel by R$ 0.46 per liter. This time, the industry action occurred with ABIR, which openly expressed its position contrary to the measure ^36^ . On September 27, 2018, the President of the Republic, Michel Temer, finally gave in to political pressure and edited Decree No. 9,514 ^37^ , restoring part of the previous subsidy and making the tax rate more favorable to the sector.

These policy processes should ideally become the subject of future academic analyses. Nevertheless, there is clear evidence of attempts by the ultra-processed products industry in Brazil to influence government decisions that could negatively affect it. This evidence reinforces the results of Mancuso’s analysis ^13^ of the Brazilian industry sector, represented by the Brazilian National Confederation of Industry: one of the forms of political action of this privileged industrial segment is to commit its resources to maintain specific advantages.

## CONCLUSION

It is true that the ultra-processed products industry, notably transnational corporations and large national companies that produce and market this type of product, holds significant economic and political power and operate as a special interest group with the aim of shaping food and nutrition public policies in its favor. Its organizational capacity and high availability of resources place the sector in a very privileged position in relation to public interest groups that advocate for collective rights, such as health and adequate food.

Despite all this, the lack of assessment of corporate influence on policy-making processes in Brazil does not allow to determine whether the sector is able to make its interests prevail. In any case, if its interest do prevail, there may be serious social consequences – including the safeguard of population health, which is a right guaranteed by the Brazilian Federal Constitution. In addition, there may be economic consequences affecting the funding of the Brazilian Unified Health System, especially with respect to actions aimed at the prevention, control, and treatment of obesity and diet-related non-communicable diseases.

Therefore, it is necessary to conduct studies seeking to determine the degree of success of the actions taken by the ultra-processed products industry, identifying the conditions associated with the convergence of the results of food and nutrition public policies with private interests . It should also be addressed to what extent the participation of civil society organizations, such as the Brazilian Alliance for Healthy and Adequate Food, is able to make public interests override private ones. It is also necessary to consider that the struggle between the different groups involved can lead to mixed results, without clear winners or losers. Finally, even if the participation of the ultra-processed products industry in public policy processes in Brazil is mainly reactive and defensive, in response to government regulatory proposals, it is important to investigate whether its search for influence is also proactively manifested, for example, by the presentation of legislative proposals by elected officials in the National Congress.
