# Supplementary material for: Ultra-processed products industry operating as an interest group
Source: Rev Saude Publica. 2020 Oct 28;54:107. doi: 10.11606/s1518-8787.2020054002127 (PMC7593022; doi:10.11606/s1518-8787.2020054002127)
Supplement: Translation

# Translation: Atuação da indústria de produtos ultraprocessados como um grupo de interesse

**Authors:** Aline Brandão Mariath, Ana Paula Bortoletto Martins

**Affiliations:** I Brasil Câmara dos Deputados. Departamento Médico. Brasília, DF, Brasil; II Brasil Universidade de São Paulo. Faculdade de Saúde Pública. Programa de Pós-Graduação em Nutrição em Saúde Pública. São Paulo, SP, Brasil; III Brasil Universidade de São Paulo. Núcleo de Pesquisas Epidemiológicas em Nutrição e Saúde. São Paulo, SP, Brasil

**Keywords:** Indústria Alimentícia, Conflito de Interesses, Política Pública, Legislação sobre Alimentos, Regulação Governamental

## Abstract

A participação da indústria de produtos ultraprocessados nos esforços para reduzir a obesidade e doenças crônicas associadas à má alimentação tem sido questionada, sobretudo porque há evidências de sua interferência no processo de formulação de políticas públicas. Este artigo se alicerça na teoria da ação coletiva e na literatura de ciência política para discutir o papel desse setor como um grupo de interesse especial que utiliza seu significativo poder econômico para influenciar decisões governamentais em seu favor. No Brasil, sua atuação ocorre principalmente por meio de associações. Ainda não se pode assegurar, todavia, que seus interesses prevaleçam no processo decisório. Sugere-se a realização de pesquisas que determinem o grau de sucesso de suas ações, identifiquem as condições associadas à convergência dos resultados das políticas com seus interesses e apontem em que medida as organizações da sociedade civil são capazes de fazer os interesses públicos se sobreporem aos privados.

## INTRODUÇÃO

Atualmente, não há dúvidas sobre a influência da indústria de produtos ultraprocessados nos ambientes alimentares e sobre sua relação com o aumento da obesidade e de doenças crônicas não transmissíveis associadas à má alimentação. Esse fenômeno é atribuído principalmente ao crescimento acelerado do consumo desses produtos. As empresas desse setor exercem grande influência nas atitudes, percepções e preferências individuais e na oferta de alimentos à população ao determinar sua disponibilidade, qualidade e preço. Além disso, utilizam-se de elaboradas estratégias de marketing para promover seus produtos e moldar normas e crenças sociais relacionadas à alimentação ^1^ . De modo geral, entre os atores que normalmente se sobressaem por direcionar as decisões alimentares de forma mais direta estão as grandes empresas nacionais e multinacionais ^2^ .

Devido ao grande alcance das principais empresas do setor alimentício, tem-se encorajado sua participação nos esforços de saúde pública para tornar os ambientes alimentares mais saudáveis. No entanto, essa atuação como parte da solução é controversa, uma vez que as ações governamentais para melhorar o sistema alimentar podem impactar os custos de produção, o crescimento das vendas e a geração de lucro. Para essas medidas serem efetivas de fato, precisam ser devidamente contextualizadas e ir além de iniciativas de autorregulação ^2^ . Nesse sentido, é necessário reconhecer que a adoção de medidas regulatórias mandatórias pelo setor público tem se mostrado essencial para proteger e promover a saúde da população e abordar adequadamente o problema da obesidade e das doenças crônicas não transmissíveis associadas à má alimentação ^5^ .

Há relatos na literatura científica de saúde pública sobre a atuação da indústria de produtos ultraprocessados para interferir no processo de formulação de políticas públicas, influenciando medidas legais e regulatórias em seu favor, ou ainda impedindo o estabelecimento de regras que impactem negativamente suas atividades ^3 , 7^ .

O objetivo deste artigo é trazer ao debate o papel da indústria de produtos ultraprocessados como um grupo de interesse especial que se utiliza de seu significativo poder econômico para intervir em decisões governamentais que lhe digam respeito. Não obstante esse tema vir ganhando cada vez mais atenção do ponto de vista da saúde pública, sua discussão sob a perspectiva da ciência política ainda é bastante incipiente.

Este ensaio se alicerça na teoria da ação coletiva, de Mancur Olson, e na revisão narrativa de literatura nacional e internacional sobre grupos de interesse. Evidências acerca da atuação da indústria de produtos ultraprocessados em processos de formulação de políticas públicas foram coletadas a partir de artigos publicados em periódicos científicos indexados nas bases de dados PubMed e SciELO, bem como em *websites* oficiais do governo federal e de associações do setor.

## DEFINIÇÕES: GRUPOS DE INTERESSE, GRUPOS DE PRESSÃO, GRUPOS DE INTERESSE ESPECIAIS E LOBBY

Existem diversas definições para grupos de interesse, algumas mais abrangentes – incluindo todos os tipos de grupos, independentemente de suas atividades, graus de organização e clivagens – e outras mais restritas, cujo foco se limita àqueles que apresentam demandas ao poder público. Quando se tornam politicamente ativos para proteger-se ou promover seus interesses, os grupos de interesse podem ser então denominados “grupos de pressão” ou “grupos de interesse especiais”, evidenciando a atividade que caracteriza sua atuação ^13^ . O termo “grupo de pressão” carrega mais conotações negativas que o termo “grupo de interesse” e parece aplicar-se apenas a grupos seccionais – aqueles que representam o interesse próprio de um grupo econômico ou social particular de uma sociedade e cujos membros a eles se restringem. Os grupos podem ainda ser considerados primários, quando seu propósito fundamental é engajar-se em atividades de *lobby* , ou secundários, quando agem politicamente apenas se necessário ^16 , 17^ .

A despeito das pequenas diferenças entre as inúmeras definições, há um consenso entre os estudiosos: grupos de interesse ou de pressão são aqueles que entram em contato com os tomadores de decisão do poder público com o objetivo de persuadi-los, influenciando as políticas públicas em seu favor. Podem ser formados por grupos de associações tradicionais, grupos de interesse institucionais ou grupos de interesse organizacionais ^13^ . Os grupos de interesse institucionais não são necessariamente organizações com membros associados, conforme o sentido tradicional de um grupo de interesse, podendo compreender instituições públicas ou privadas – inclusive empresas individuais –, e os grupos de interesse organizacionais podem incluir associações que representem seus membros ^15^ .

No tocante à atuação das empresas como grupos de interesse, essas podem ser consideradas um grupo de interesse especial ^18^ e normalmente se articulam diretamente ou por meio de representantes sempre que há a percepção de que decisões do poder público podem interferir em suas atividades ^13^ . De acordo com Olson ^19^ , os interesses especiais são altamente organizados, apresentam poder político desproporcional em relação às demais organizações e tendem a prevalecer sobre interesses não organizados de grandes grupos. Assim, pequenos grupos formados por grandes unidades empresariais utilizam-se de sua capacidade de ação organizada e voluntária para criar um *lobby* ativo, exercer pressão e obter então vantagens políticas. Resumem-se as principais definições relativas aos grupos de interesse no Quadro .

**Quadro:** Resumo das definições e dos tipos de grupos de interesse.

Definições	
Grupos de pressão	Grupos de interesse que entram em contato com os tomadores de decisão do poder público com o objetivo de persuadi-los a influenciar as políticas públicas em seu favor. Este termo normalmente se aplica a grupos seccionais (que representam o interesse próprio de um grupo econômico ou social particular e cujos membros a eles se restringem).
Grupos de interesse especiais	Grupos de interesse altamente organizados, normalmente com desproporcional poder econômico (e, consequentemente, político) em relação às demais organizações.
**Tipos**	
Primários	Grupos de interesse cujo propósito fundamental é engajar-se em atividades de *lobby* .
Secundários	Grupos de interesse cujo propósito fundamental não é político, mas que podem agir de tal forma se necessário.
Institucionais	Compreendem instituições públicas ou privadas (incluindo empresas individuais).
Organizacionais	Compreendem associações comerciais que representam seus membros.

Os conceitos e teorias apresentados acima se aplicam diretamente à indústria de produtos ultraprocessados, especialmente porque se trata de um setor concentrado, em escala global, em um pequeno grupo de empresas transnacionais com grande poder econômico. Tem-se então um grupo de interesse especial, que pode ser tanto primário, no caso das empresas consideradas individualmente, quanto secundário, quando atuam por meio de suas associações.

O *lobby* é a modalidade típica de atuação dos grupos de interesse nos sistemas pluralistas, em que as diversas organizações que reivindicam a representação de seus interesses buscam influenciar o processo decisório do poder público ^20^ . Trata-se de um processo multifacetado que inclui coleta de informações, preparação de projetos de política, definição de estratégias para defesa desses projetos, busca por aliados, entre outras atividades. Envolve também o exercício de pressão, mas somente no seu último estágio. O lobista, apesar de representar interesses especiais, detém informações e conhecimentos técnicos e políticos especializados, e frequentemente se mostra útil na definição da legislação e da regulamentação ^21^ . Embora o *lobby* lícito possa ser positivo, pois aproxima o resultado decisório das preferências dos interesses organizados, pode ter consequências indesejáveis quando há grande desequilíbrio de forças entre os grupos, de modo que aqueles mais fortes e organizados são excessivamente privilegiados, podendo colocar em xeque o interesse público ^20^ .

## DESIGUALDADE DE REPRESENTAÇÃO E DE RECURSOS ENTRE OS GRUPOS DE INTERESSE

Os grupos de interesse são centrais para a representação política, a agenda de políticas públicas e os resultados dessas políticas. Todavia, há vieses importantes no sistema de representação. Grupos privilegiados, como os empresariais, são normalmente super-representados nas arenas políticas, enquanto grupos de interesse público, que buscam principalmente o bem coletivo (aquele que quando alcançado não beneficia seus membros ou ativistas de forma seletiva ou material), frequentemente enfrentam mais dificuldades para se organizar e são normalmente sub-representados ^22^ . Ademais, o exercício de influência sobre as autoridades políticas nas democracias modernas exige um conjunto de competências que inclui a capacidade de superar problemas relacionados à ação coletiva, mobilizar recursos, desenvolver *expertise* , manter atenção contínua, coordenar ações com outros atores e agir nos mais variados contextos ^23^ .

A promoção de interesses nas instâncias decisórias do poder público depende em grande medida da disponibilidade de recursos. Sem dúvida, o mais importante deles é o dinheiro, visto que viabiliza as atividades de defesa de interesses e consequentemente aumenta a efetividade das intervenções dos grupos ^20 , 24^ . Grandes empresas apresentam vantagens nesse ponto: com frequência, sua folga organizacional permite a realização de atividades que não tenham efeito imediato sobre seus lucros e, por disporem de mais recursos financeiros, conseguem empregar estratégias políticas mais sofisticadas ^18^ .

Conquanto o dinheiro seja o recurso mais importante, há inúmeros outros que podem ser utilizados, com destaque para a legitimidade ou afinidade com as preferências da opinião pública, a dimensão do segmento social representado e a proporção do segmento efetivamente perfilada atrás dos *lobbies* , assim como as características dos lobistas ^20^ . Também é importante notar que a comunidade de negócios não necessariamente intervém politicamente da mesma forma ou com o mesmo grau de frequência ou intensidade, podendo direcionar estrategicamente seus recursos para lugares e situações que produzirão resultados mais eficientes ^24^ .

O acesso às arenas políticas é condição necessária para o exercício da influência na agenda e nas decisões desse âmbito, ainda que por si só não seja suficiente. A oferta de recursos é importante porque as instituições do Estado e os grupos de interesse mantêm uma relação de interdependência, posto que não são capazes de alcançar seus objetivos políticos de forma autônoma. Entretanto, observa-se que o acesso a uma arena abre portas para acessar outras arenas políticas, em um efeito cascata e cumulativo. Assim, alguns atores, por deterem mais recursos, são capazes de dominar múltiplas arenas, caracterizando o “pluralismo privilegiado” ^22^ .

Para Mancuso e Gozetto ^20^ , se houvesse equilíbrio entre os grupos atuantes, o *lobby* lícito poderia proporcionar contribuições positivas para os interesses defendidos e os tomadores de decisão, colaborando para a legitimação do sistema político. Dentre as contribuições dos grupos para a democracia, segundo os pluralistas, destacam-se a oferta de informações especializadas para o governo, contribuindo para a inclusão de itens importantes na agenda política, e a melhora da qualidade do debate público, canalizando as opiniões para a mídia ^17^ . Contudo, os recursos não estão amplamente disponíveis e distribuídos equitativamente entre os grupos de interesse e, portanto, o acesso ao processo de formulação de políticas e as chances de influenciá-las não são igualitários ^18 , 24^ . De fato, nenhum grupo de interesse – tal como sindicatos ou grupos de interesse público – é capaz de competir com os volumosos recursos empresariais ^18^ . Logo, pode-se afirmar que a desigualdade de representação e de recursos tem como consequência a desigualdade de acesso e de participação no processo de políticas públicas. Além disso, embora essas desigualdades reflitam o viés geral da distribuição de poder, elas podem estar simplesmente não o replicando, mas reforçando-o ^16^ .

Outro ponto importante a ser considerado, destacado por Coen et al. ^18^ , é a relação de poder entre o Estado e as empresas, denominada pelos autores de “estruturalismo”: os Estados dependem fundamentalmente das empresas, pois necessitam dos recursos e receitas por elas gerados. Assim, se por um lado o governo pode controlar as empresas, por outro, também pode ser por elas controlado e dominado, no que se denomina captura do Estado. O estudo das relações entre empresas e Estados suscita questões acerca da efetividade do governo, da democracia e da distribuição do poder nas sociedades modernas, principalmente porque grupos organizados que detêm mais poder podem dominar e ajudar diretamente a criar iniquidades ^18 , 24^ . Portanto, apesar de fazerem parte dos sistemas políticos, os grupos de interesse, ao promover benefícios enviesados em favor de alguns segmentos e em detrimento de outros, podem prejudicar os objetivos fundamentais de uma sociedade. Ressalta-se que, embora seja defendida a necessidade de regulação das atividades dos grupos de interesse, mesmo que esta seja capaz de afetar o seu acesso, não necessariamente impactará seu poder de influência ou reduzirá a desigualdade ^15^ .

## OBJETIVOS, ESTRATÉGIAS E CONSEQUÊNCIAS DA ATUAÇÃO DOS GRUPOS DE INTERESSE

Os grupos de interesse não buscam o exercício direto do poder, mas influenciar direta ou indiretamente os processos políticos nas questões específicas que lhes dizem respeito. Sua interação com os formuladores de políticas públicas normalmente ocorre por meio de *lobby* , cujas atividades nem sempre buscam uma influência imediata sobre o processo decisório, podendo ser empregadas apenas com o intuito de facilitar o acesso aos tomadores de decisão e construir um relacionamento com representantes do governo com vistas à concessão de benefícios no futuro ^15^ .

A participação dos grupos de interesse nos processos políticos pode ocorrer em diversas de suas etapas, desde a definição da agenda até a formulação e implementação das políticas ^17 , 22^ . Sabe-se que os grupos frequentemente atuam em coalizão ou competição em determinadas áreas e empregam uma ampla variedade de táticas para moldar as políticas públicas ^24 , 25^ . Porém, analisar os efeitos causais de suas intervenções é uma tarefa bastante complexa, dada a multiplicidade de atores, recursos e estratégias envolvidos, como também o fato de que a maior parte das ações acontece longe do escrutínio público.

Apesar da dificuldade de se comprovar a relação de causa e efeito entre o *lobby* e o resultado da política, especialmente porque circunstâncias econômicas, socioculturais e históricas também interferem no cálculo do decisor, é possível mensurar o sucesso do lobista ^20^ . Mancuso ^26^ considera haver sucesso político quando o teor da decisão converge com a posição empresarial, podendo ocorrer tanto em situações em que a decisão do setor público melhora o *status quo* como quando uma decisão que levaria à sua piora não é tomada. Já o insucesso político, assim considerado quando há divergência entre o teor da decisão pública e a posição empresarial, ocorre em dois casos: quando o empresário ou seus representantes lutam contra uma decisão que piorará o *status quo* , mas a decisão é tomada mesmo assim; e quando o empresariado defende uma decisão que melhoraria o *status quo* , mas esta não é tomada pelo poder público.

Nesse contexto, pode-se então afirmar que a atuação política do empresariado pode ter dois objetivos: 1) melhorar o *status quo* atual, o que demanda uma atitude ofensiva; e 2) manter ou impedir a piora do *status quo* , em uma atitude defensiva ^26^ . Assim, os grupos de interesse podem agir tanto ofensiva e pró-ativamente em busca de favorecimento em políticas públicas como de forma defensiva e reativa, buscando a atenuação de medidas desfavoráveis. Sobretudo no que concerne à atitude defensiva, Hacker e Pierson ^23^ chamam a atenção para uma estratégia bastante explorada pelos grupos de interesse: encorajar a não ação por parte dos representantes do governo, dado que isso não atrai a atenção dos eleitores e do público em geral, tampouco pode ser atribuído a um formulador de política pública em particular.

Geralmente, há diferentes preferências dos grupos de interesse no emprego de recursos, na busca pelo acesso às arenas políticas e na definição dos alvos e pontos de pressão. Grupos empresariais costumam priorizar o uso de *insider resources* – tais como informações e *expertise* relevantes para o processo decisório – e atuar em arenas menos visíveis, principalmente quando o objetivo político é influenciar a tomada de decisões ^17 , 22^ .

No que tange à indústria de produtos ultraprocessados, não necessariamente sua articulação de interesses se limita à abordagem dos tomadores de decisão. Quando engajado em atividade política corporativa, o setor emprega uma multiplicidade de estratégias, entre elas: a produção e disseminação de informações favoráveis às suas atividades; a distribuição de incentivos, inclusive financeiros, para políticos, partidos políticos e tomadores de decisão; o estímulo à formação de opinião pública favorável à empresa; a desestabilização de grupos ou indivíduos que criticam ou se opõem a seus produtos ou práticas, ou ainda que defendem políticas que podem impactar negativamente seus negócios; e o uso ou a ameaça de uso de processos judiciais, seja para barrar decisões governamentais que lhe sejam desfavoráveis, seja para intimidar seus opositores ^12^ . Recentemente, foi demonstrado ainda que o modelo denominado de *policy dystopia* , desenvolvido para descrever a atividade política corporativa da indústria do tabaco – amplamente reconhecida por sua ação intensa para minar os esforços em saúde pública no sentido de reduzir os malefícios causados à população pelo uso de seus produtos –, também pode ser aplicado à indústria de produtos ultraprocessados ^27 , 28^ .

## EVIDÊNCIAS DA ATUAÇÃO DA INDÚSTRIA DE PRODUTOS ULTRAPROCESSADOS COMO GRUPO DE INTERESSE NO BRASIL

No Brasil, ainda há uma importante lacuna de conhecimento no que diz respeito à atuação da indústria de produtos ultraprocessados para influenciar as políticas públicas de alimentação e nutrição. Do ponto de vista acadêmico, até o momento temos conhecimento apenas de análises sobre o processo de regulação de publicidade de alimentos pela Agência Nacional de Vigilância Sanitária (Anvisa), iniciado em 2005 e que resultou na Resolução da Diretoria Colegiada (RDC) nº 24/2010. De acordo com Henriques et al. ^29^ , o setor regulado não escondeu seu posicionamento contrário à versão preliminar da norma e sua preferência pela manutenção do *status quo* . Baird ^30^ examinou a ação política dos grupos de interesse empresariais nesse processo, em que ficou evidente sua intervenção na Anvisa e nos Poderes Executivo e Legislativo para influenciar os resultados da política pública, intensificada após a publicação da norma em questão. Para o autor, o acesso privilegiado dos lobistas, facilitado pelo grande poder econômico da indústria, contribuiu para a elaboração de uma versão mais branda do regulamento. Como resultado, na redação final da norma foram excluídos elementos relacionados à publicidade infantil que constavam na proposta inicial, tornando-a menos restritiva ^29^ .

Chama a atenção nesse caso o fato de não ter havido manifestação direta – favorável ou contrária – de nenhuma empresa de produtos ultraprocessados durante o processo de construção da norma. Sua participação se deu exclusivamente de forma indireta, por meio da Associação Brasileira da Indústria de Alimentos (Abia) e da Associação Brasileira das Indústrias de Refrigerantes e de Bebidas Não Alcoólicas (Abir) ^29 , 31^ . Isso corrobora a literatura que aponta que a atuação coletiva é vantajosa principalmente quando há interesses comuns em jogo ou os atores envolvidos são ameaçados por políticas regulatórias. Essas políticas atingem todo o setor industrial e tendem a desencadear maior nível de conflito, frequentemente estimulando os atores afetados tanto a atuar individualmente quanto a formar coalizões para enfrentar aqueles que defendem interesses diferentes dos seus ^14 , 19 , 21 , 26 , 32^ .

Atualmente, há uma série de evidências empíricas sobre a atuação intensa da indústria de produtos ultraprocessados em dois processos regulatórios em andamento. No primeiro caso, de processo iniciado em 2014 pela Anvisa com o objetivo de estabelecer novas regras para a rotulagem nutricional de alimentos industrializados, o setor tem sido representado tanto pela Abia quanto por um conjunto de outras associações reunidas na Rede Rotulagem. Esses atores têm utilizado diversas estratégias para moldar o debate e favorecer a adoção de uma norma que tenha menor impacto sobre as escolhas dos consumidores e, portanto, seja mais favorável ao setor: o modelo de rotulagem nutricional do semáforo. Dentre essas, pode-se citar inclusive o uso de uma estratégia legal, com a obtenção, pela Abia, de uma liminar do Poder Judiciário para prorrogar a tomada pública de subsídios aberta pela Anvisa em 2018, sugerindo uma tentativa de retardar o processo decisório ^33^ . Considerando a divulgação do Relatório Preliminar de Análise de Impacto Regulatório sobre Rotulagem Nutricional ^34^ , o qual, a partir da avaliação das evidências e contribuições geradas na tomada pública de subsídios, apontou favorecimento do modelo frontal de advertência quanto à presença de nutrientes críticos em excesso, é esperado que a indústria de produtos ultraprocessados reforce sua oposição e amplie o uso de estratégias para tentar barrar a regulação e/ou influenciar a decisão final da Agência.

O segundo caso refere-se aos desdobramentos do Decreto nº 9.394, de 30 de maio de 2018 ^35^ , que retirou parte dos benefícios fiscais concedidos por meio de créditos no imposto sobre produtos industrializados às empresas que produzem concentrados de refrigerantes na Zona Franca de Manaus. Essa foi uma das medidas adotadas pelo Governo Federal para compensar a queda na arrecadação de impostos gerada após a greve dos caminhoneiros, encerrada mediante o compromisso do Estado de reduzir o preço final do diesel em R$ 0,46 por litro. Dessa vez, a atuação da indústria se deu por intermédio da Abir, que manifestou abertamente seu posicionamento contrário à medida ^36^ . Em 27 de setembro de 2018, o presidente da República, Michel Temer, finalmente cedeu à pressão política e editou o Decreto nº 9.514 ^37^ , restabelecendo parte do subsídio anterior e tornando a alíquota do imposto um pouco mais favorável ao setor.

Esses processos, idealmente, devem tornar-se objeto de análises acadêmicas futuras. De qualquer forma, há evidências claras de tentativas por parte da indústria de produtos ultraprocessados no país de influenciar decisões governamentais que lhe possam trazer algum tipo de prejuízo. Essas evidências reforçam os resultados da análise de Mancuso ^13^ sobre a atuação do empresariado industrial brasileiro, representado pela Confederação Nacional da Indústria: uma das formas de atuação política desse segmento industrial privilegiado é empenhar seus recursos com vistas à manutenção de vantagens particulares.

## CONSIDERAÇÕES FINAIS

É certo que a indústria de produtos ultraprocessados, notadamente as empresas transnacionais e grandes empresas nacionais que produzem e comercializam esse tipo de produto, detém importante poder econômico e político e atua como um grupo de interesse especial com o objetivo de moldar as políticas públicas de alimentação e nutrição em seu favor. Sua capacidade de organização e sua elevada disponibilidade de recursos colocam o setor em posição bastante privilegiada em relação principalmente a grupos de interesse público que fazem *advocacy* por diretos coletivos, como a saúde e a alimentação adequada.

A despeito disso, a escassez de avaliações sobre sua influência no processo de tomada de decisões públicas no Brasil não permite determinar se o setor consegue fazer prevalecer seus interesses. De toda forma, caso isso ocorra, pode haver sérias consequências sociais – com prejuízo à garantia do direto à saúde pelo Estado, assegurado pela Constituição Federal de 1988 ^38^ – e econômicas, com impacto nos custos de financiamento do Sistema Único de Saúde, em especial no que se refere às ações de prevenção, controle e tratamento da obesidade e de doenças crônicas não transmissíveis associadas à má alimentação.

Faz-se necessária, então, a realização de estudos que busquem determinar o grau de sucesso do setor de produtos ultraprocessados em suas ações, identificar as condições associadas à convergência dos resultados das políticas públicas voltadas a alimentação e nutrição com os interesses da indústria e em que medida a atuação de organizações da sociedade civil – entre elas a Aliança pela Alimentação Adequada e Saudável – é capaz de fazer os interesses públicos se sobreporem aos privados. É preciso considerar ainda que o embate entre os diferentes grupos envolvidos pode acarretar resultados mistos, sem vencedores ou perdedores claros. Por fim, mesmo que a atuação da indústria de produtos ultraprocessados no Brasil pareça se dar de forma reativa e defensiva, como resposta a propostas de regulamentação governamental, é importante investigar se sua busca por influência também se manifesta de forma proativa, por exemplo, por meio da apresentação de proposições legislativas por parlamentares no Congresso Nacional.
